# Case Report: Multiple anterior chamber paracentesis or washout for a large hyphema following vitrectomy with silicone oil tamponade

**DOI:** 10.3389/fopht.2025.1621845

**Published:** 2025-12-03

**Authors:** Jing Li, Jie Peng, Qian Luo, Wanqiu Zhang, Yao Yao, Shiyan Chen, Lin Zhou

**Affiliations:** Department of Ophthalmology, Sichuan Provincial People’s Hospital, University of Electronic Science, Technology of China, Chengdu, China

**Keywords:** hyphema, pars plana vitrectomy, anterior chamber paracentesis, anterior chamber washout, silicone oil tamponade

## Abstract

**Introduction:**

To report the management of a long-standing, large hyphema following vitrectomy with silicone oil tamponade using multiple anterior chamber paracentesis (ACP) or washout (ACW).

**Case presentation:**

A 50-year-old woman presented with blurred vision attributed to polypoidal choroidal vasculopathy and vitreous hemorrhage. Vitrectomy with silicone oil tamponade and lens extraction were performed. A persistent large hyphema and elevated intraocular pressure were detected postoperatively. ACP and ACW were performed five and two times, respectively. The hyphema faded, and the elevated IOP decreased to the normal range within 2 months. The cornea was clear, and corneal blood staining was not observed.

**Conclusions:**

Multiple ACPs and ACWs are necessary and effective for the treatment of long-standing large hyphema.

## Introduction

The accumulation of red blood cells in the space between the cornea and iris is referred to as hyphema. A history of trauma or recent ocular surgery is the most common risk factor (1). In addition to the direct consequences of the initial injury, hyphema is considered a self-limiting condition that rarely causes permanent loss of vision in the absence of associated damage to the cornea, lens, or optic nerve. Hyphema complications can result in irreversible vision loss, including glaucoma, corneal blood staining, and optic atrophy, especially in cases of prolonged hyphema in association with elevated intraocular pressure (IOP) (2).

Hyphema is rarely encountered but influences surgical outcomes and occurs in patients following intraocular surgeries (3, 4). Postoperative hyphema following vitrectomy with silicone oil tamponade is uncommon, with an incidence ranging from 1% to 16.8% (5, 6). The mechanisms are multifactorial. Possible causes include (1) migration of liquefied blood from the posterior segment through zonular or capsular defects under the buoyant force of silicone oil; (2) rupture of fragile iris or angle vessels due to intraoperative manipulation, postoperative inflammation, or IOP fluctuation; (3) direct trauma to iris or ciliary body vessels during surgery; (4) silicone oil–related inflammation disrupting the blood–aqueous barrier; and (5) delayed liquefaction of preretinal clots allowing anterior migration of blood (3–5). These mechanisms have been described in association with fibrovascular membrane removal and intraoperative bleeding after vitrectomy (6).

Herein, we report a case of a 50-year-old woman with decreased visual acuity in her right eye owing to vitreous hemorrhage attributed to polypoidal choroidal vasculopathy (PCV). A persistent large hyphema was detected following vitrectomy with silicone oil tamponade. Hyphema was faded after six times of anterior chamber paracentesis (ACP) and two times of anterior chamber washout (ACW).

## Informed consent

Informed consent was obtained from the patient prior to participation in the study. The research was conducted in accordance with the principles outlined in the Declaration of Helsinki and was approved by the Ethics Committee of the hospital.

## Case description

A 50-year-old woman complained of decreased visual acuity in her right eye for 1 month. The patient had a history of hypertension for 12 years. Fundus examination revealed a subretinal hemorrhage ranging from the superior temporal region of the optic disc to the macula. Polyps and branching vascular network were detected by optical coherence tomography angiography, which revealed PCV. Visual acuity was 20/63, and IOP was 13.2 mmHg. Ranibizumab was administered by intravitreal injection 3 days later.

**Figure 1:**
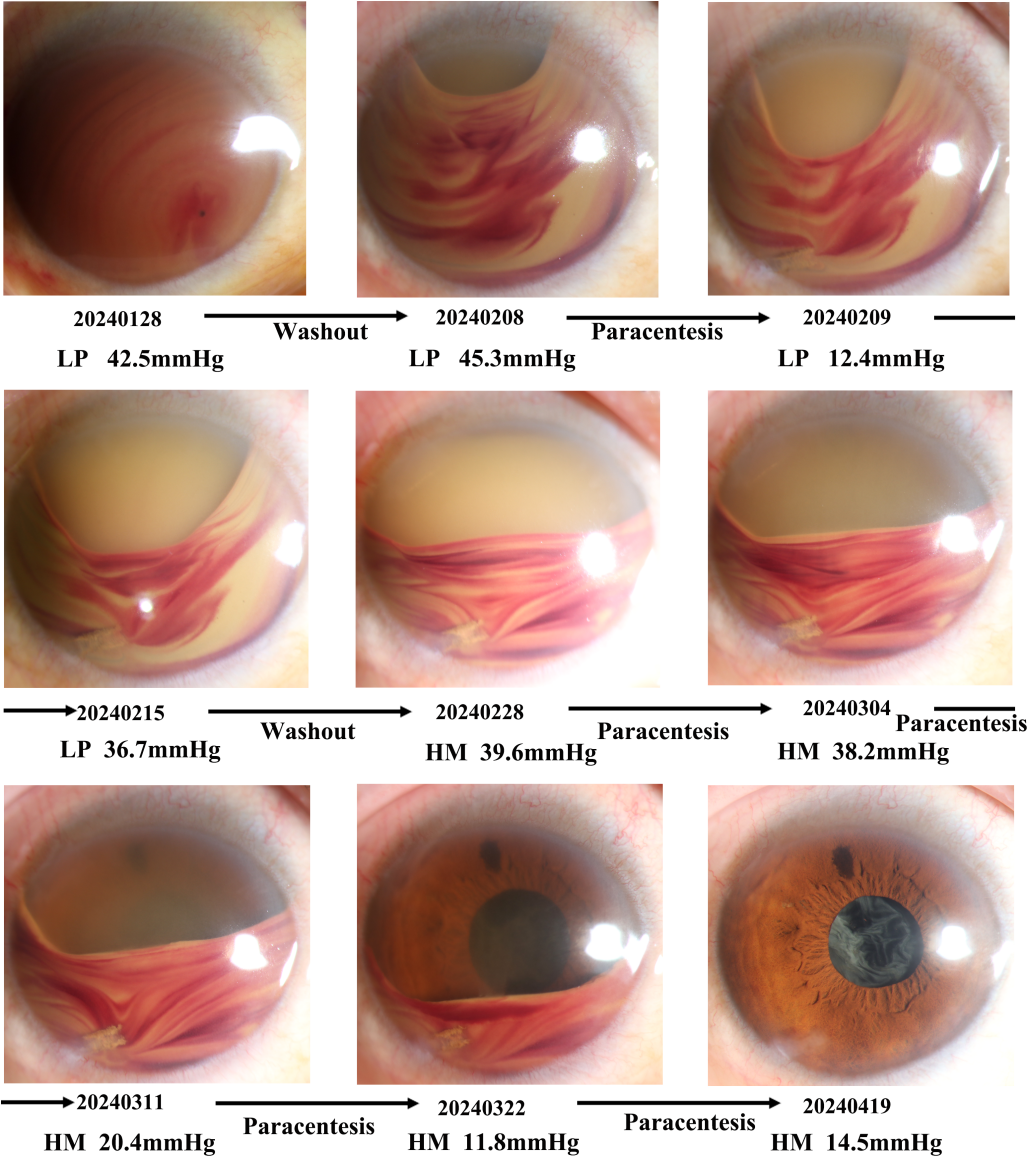
Timeline of the treatments and response for hyphema. The figure shows the state of the anterior segment at each stage of treatment. At the bottom of the anterior segment, photography, timepoint, visual acuity, and intraocular pressure were listed.

The patient experienced a sudden decrease in vision (hand moving), and vitreous hemorrhage was detected approximately 1 week later after the injection. The vitreous hemorrhage did not resolve after 2 weeks of observation, and the vision did not improve. Vitrectomy with silicone oil tamponade and lens extraction were performed immediately. Headache, vomiting, eye pain, and low vision were observed in the patient 1 week after the surgery. Hyphema was appeared, and blood mixed with silicone oil filled the total anterior chamber (Grade IV hyphema) (01/28/2024). IOP increased to 52.3 mmHg, and visual acuity decreased to light perception. Mannitol, beta-blockers, alpha agonists, and carbonic anhydrase inhibitors were used to control the increased IOP. However, no obvious change in the hyphema was detected, and elevated IOP persisted. When anterior chamber washout and clot removal were performed for the first time (1/30/2024), the IOP decreased to 18.1 mmHg, and the hyphema disappeared the next day (1/31/2024). However, the visual acuity remained the same. One week later (02/08/2024), hyphema was observed in the entire anterior chamber again, and the IOP increased to 45.3 mmHg. ACP was performed on two consecutive days (02/08/2024 and 02/09/2024). Part of the blood mixed with silicone oil flowed out, and the IOP decreased to 12.4 mmHg. Approximately 1 week after the follow-up, the height of the hyphema was evidently reduced, and the IOP decreased (36.7 mmHg) (02/15/2024). However, the blood mixed with silicone oil filled one-half to less than the total volume of the anterior chamber (Grade III hyphema). To eliminate the hyphema as quickly as possible, the patient underwent anterior chamber washout once again (02/18/2024). 10 days after anterior chamber washout, hyphema and elevated IOP (39.6 mmHg) were observed (02/28/2024), while the anterior chamber washout demonstrated a good clinical outcome and the accumulated blood filled only half of the anterior chamber. ACP was performed for the third time. The color of the anterior chamber faded 1 week later. However, the height of the hyphema remained the same and the IOP was still high (38.2 mmHg) (03/04/2024). When ACP was performed for the fourth time, the color of the anterior chamber faded, the iris was visible 1 week later, and the IOP was normal (20.4 mmHg). The height of the hyphema decreased compared with that during the fifth paracentesis (03/11/2024). Upon performing ACP for the sixth time, the inferior space of the anterior chamber and the structure of the iris appeared very clear, and the IOP decreased to the normal range (11.8 mmHg) (03/22/2024). Upon completion of the ACP procedure, the patient returned for follow-up after 2 months. The hyphema disappeared, and exudative membranes of the pupil and corneal macula were evident (04/19/2024) (**Figure 1**).

No abnormality was observed in the left eye, and the fundus was not clear following fading of the hyphema. Yttrium-aluminum-garnet capsulotomy was recommended to eliminate the exudative membrane of the pupil and posterior capsule opacification. Further clinical data are not available as the patient did not return for follow-up.

## Discussion

Persistent high-grade hyphema and elevated IOP were observed in a 50-year old woman with PCV and vitreous hemorrhage following vitrectomy with silicone oil tamponade and lens extraction. Persistent high-grade hyphema gradually resolved after two times of anterior chamber washout six times of anterior chamber paracentesis.

Hyphema is rarely encountered but influences surgical outcomes and occurs in patients following intraocular surgeries (3, 4). The incidence of postoperative hyphema ranges from 1% to 16.8% after vitrectomy (5, 6). The management of hyphema ranges from conservative to surgical intervention, aiming to accelerate blood absorption and prevent complications. Low-grade hyphema is self-limiting and can resolve within 1 week. Bed rest or activity restriction can decrease the risk of secondary hemorrhage. Eye patching is encouraged to prevent further trauma to the injured eye. Anticoagulant and antiplatelet medications should be discontinued to prevent persistent or secondary hemorrhage. Mydriatics or cycloplegics could be administered for 2 weeks to reduce the risk of posterior synechiae. Corticosteroids, which reduce the formation of posterior synechiae and decrease the frequency of rebleeding, may stabilize the blood-ocular barrier (7). Elevated IOP can be managed with beta-blockers, alpha-agonists, carbonic anhydrase inhibitors, or systemic osmotic agents (3).

Effective control of IOP is essential to preventing vision-threatening complications such as secondary glaucoma and corneal blood staining. Persistent IOP elevation can cause ischemic and mechanical injury to the optic nerve, while prolonged contact of blood with the corneal endothelium leads to hemoglobin and iron deposition, resulting in irreversible corneal staining. Therefore, prompt IOP reduction and early evacuation of intraocular blood are crucial to protecting both the optic nerve and corneal endothelium. Approximately 5%–7.2% of patients with hyphema require surgical intervention, particularly those with total (eight-ball) hyphema, persistent high IOP, or corneal blood staining refractory to medical therapy.

Surgical options for managing hyphema include ACP, anterior chamber washout and clot removal, trabeculectomy, and peripheral iridectomy (7). In this case, a sequential and minimally invasive approach using repeated ACPs and ACWs was adopted instead of early trabeculectomy or silicone oil exchange. This conservative strategy effectively lowered IOP and cleared the anterior chamber while avoiding complications such as hypotony, infection, and retinal detachment associated with more aggressive procedures like trabeculectomy or early oil removal (8).

All ACPs were performed with a 27-gauge or 30-gauge needle under sterile conditions, inserted tangentially through the limbal cornea with the bevel up to allow controlled aqueous drainage under continuous IOP monitoring. Its safety has been well established under slit-lamp guidance, with complication rates of 0.7%–7%. Most reported complications are minor, including mild hyphema and transient hypotony (9, 10). Severe complications such as iris trauma, profound hypotony, keratitis, or endophthalmitis are rare (11).

Our report describes a single clinical case, and the findings should be interpreted cautiously. Larger studies and longer follow-up are needed to confirm the safety, efficacy, and long-term outcomes of repeated anterior chamber paracentesis and washout in similar postoperative settings.

In summary, a long-standing, large hyphema and high IOP were observed after vitrectomy with silicone oil tamponade for PCV. ACP and anterior chamber washout were performed six and two times, respectively. The hyphema gradually faded, and the IOP was decreased to the normal range. Multiple anterior chamber paracentesis or washout is safe and effective for large long-standing and large hyphema.

## Data Availability

The original contributions presented in the study are included in the article/supplementary material. Further inquiries can be directed to the corresponding author.
